# DIALIGN-TX: greedy and progressive approaches for segment-based multiple sequence alignment

**DOI:** 10.1186/1748-7188-3-6

**Published:** 2008-05-27

**Authors:** Amarendran R Subramanian, Michael Kaufmann, Burkhard Morgenstern

**Affiliations:** 1 University of Tübingen, Wilhelm-Schickard-Institut für Informatik, Sand 13, 72076 Tübingen, Germany; 2 University of Göttingen, Institute of Microbiology and Genetics, Goldschmidtstr. 1, 37077 Göttingen, Germany

## Abstract

**Background:**

DIALIGN-T is a reimplementation of the multiple-alignment program DIALIGN. Due to several algorithmic improvements, it produces significantly better alignments on locally and globally related sequence sets than previous versions of DIALIGN. However, like the original implementation of the program, DIALIGN-T uses a a straight-forward greedy approach to assemble multiple alignments from local pairwise sequence similarities. Such greedy approaches may be vulnerable to spurious random similarities and can therefore lead to suboptimal results. In this paper, we present DIALIGN-TX, a substantial improvement of DIALIGN-T that combines our previous greedy algorithm with a progressive alignment approach.

**Results:**

Our new heuristic produces significantly better alignments, especially on globally related sequences, without increasing the CPU time and memory consumption exceedingly. The new method is based on a guide tree; to detect possible spurious sequence similarities, it employs a vertex-cover approximation on a conflict graph. We performed benchmarking tests on a large set of nucleic acid and protein sequences For protein benchmarks we used the benchmark database BALIBASE 3 and an updated release of the database IRMBASE 2 for assessing the quality on globally and locally related sequences, respectively. For alignment of nucleic acid sequences, we used BRAliBase II for global alignment and a newly developed database of locally related sequences called *DIRM-BASE 1*. IRMBASE 2 and DIRMBASE 1 are constructed by implanting highly conserved motives at random positions in long unalignable sequences.

**Conclusion:**

On BALIBASE3, our new program performs significantly better than the previous program DIALIGN-T and outperforms the popular global aligner CLUSTAL W, though it is still outperformed by programs that focus on global alignment like MAFFT, MUSCLE and T-COFFEE. On the locally related test sets in IRMBASE 2 and DIRM-BASE 1, our method outperforms all other programs while MAFFT E-INSi is the only method that comes close to the performance of DIALIGN-TX.

## 1 Introduction

DIALIGN is a widely used software for multiple alignment of nucleic acid and protein sequences [1,2] that combines local and global alignment features. Pairwise or multiple alignments are composed by aligning local pairwise similarities. More precisely, pairwise local gap-free alignments called *fragment alignments *or *fragments *are used as building blocks to assemble multiple alignments. Each possible fragment is given a score that is related to the *P values *used by BLAST [3,4], and the program then tries to find a *consistent *set of fragments from all possible sequence pairs, maximizing the total score of these fragments. Gaps are not penalized. Here, *consistency *means that a set of fragment alignments can be included into one single alignment without contradictions, see for example [5] for a more formal definition of our notion of consistency.

The main difference between DIALIGN and more traditional alignment approaches is the underlying *scoring scheme *or *objective function*. Instead of summing up substitution scores for aligned residues and subtracting gap penalties, the score of an alignment is based on P-values of local sequence similarities. Only those parts of the sequences are aligned that share some statistically significant similarity, unrelated parts of the sequences remain unaligned. This way, the method can produce global as well as local alignments of the input sequences, whatever seems more appropriate. Combining local and global alignment features is particularly important if genomic sequences are aligned where islands of conserved homologies may be separated by non-related parts of the sequences. Thus, DIALIGN has been used for comparative genomics [6], for example to find protein-coding genes in eukaryotes [7-9].

As with traditional objective functions for sequence alignment, numerically optimal *pairwise *alignments can be calculated efficiently in the segment-based approach. In DIALIGN, this is done by a space-efficient fragment-chaining algorithms [10,11]. However, it is computationally not feasible to find mathematically optimal *multiple *alignments. Thus, heuristics must be used if more than two sequences are to be aligned. All previous versions of DIALIGN used a *greedy *algorithm for multiple alignment. In an initial step, optimal pairwise alignments are calculated for all possible pairs of input sequences. Since these pairwise alignments are completely independent of each other, they can be calculated on parallel processors [12]. Fragments from these pairwise alignments are then sorted by their scores, i.e. based on their P-values, and then included one-by-one into a growing *consistent *set of fragments involving all pairs of sequences – provided they are consistent with the previously included fragments.

The greedy algorithm used in DIALIGN is vulnerable to spurious random similarities. It has been shown, that the numerical score of alignments produced by this heuristic can be far below the optimum [5,13]. Consequently, alternative optimization algorithms have been applied to the optimization problem defined by the DIALIGN approach, e.g. *Integer Linear Programming *[14].

## 2 Assembling alignments from fragments

Formally, we consider the following optimization problem: we are given a set *S *= {*s*_1_, . . ., *s*_*k*_} of input sequences where *l*_*i *_is the length of sequence *s*_*i*_. A fragment *f *is a pair of two equal-length segments from two different input sequences. Thus, a fragment represents a local pairwise gap-free alignment of these two sequences. Each possible fragment *f *is assigned a *weight score w*(*f*) which, in our approach, depends on the probability *P*(*f*) of *random occurrence *of such a fragment. More precisely, if *f *is a local alignment of sequences *s*_*i *_and *s*_*j*_, then *P *(*f*) is the probability of finding a fragment of the same length as *f *with at least the same sum of matches or similarity values for amino acids in *random *sequences of length *l*_*i *_and *l*_*j*_, respectively. For protein alignment, a standard substitution matrix is used. Let *F *be the set of all possible fragments. The optimization problem is then to find a *consistent *set *A *⊂ *F *of fragments with maximum total weight, i.e. a consistent set *A *maximizing

$$W(A):=\sum_{f\in A}w(f).$$

A set of fragments is called *consistent *if all fragments can be included into one single alignment, see [15]. Fragments in *A *are allowed to overlap if different pairs of sequences are involved. That is, if two fragments *f*_1_, *f*_2_∈ *A *involve sequence pairs *s*_*i*_, *s*_*j *_and *s*_*j*_, *s*_*k*_, respectively, then *f*_1 _and *f*_2 _are allowed to overlap in sequence *s*_*j*_. If two fragments involve the same pair of sequences, no overlap is allowed. It can be shown that the problem of finding an optimal consistent set *A *of fragments is NP-complete (Constructing multiple sequence alignments from pairwise data, Subramanian *et al*., in preparation). Therefore, we are motivated in finding intelligent approximations that deliver a good tradeoff between alignment quality and CPU time.

To decrease the computational complexity of this problem, we restrict ourselves to a reduced subset *F' *⊂ *F *and we will first search for a consistent subset *A *⊂ *F' *with maximum total score. As in previous versions of DIALIGN, we use pairwise optimal alignments as a filter. In other words, the set *F' *is defined as the set of all fragments contained in any of the optimal *pairwise *alignments of the sequences in our input data set. Here, we also restrict the length of fragments using some suitable constant.

For multiple alignment, previous versions of DIALIGN used the above outlined *greedy *approach. We call this approach a *direct *greedy approach, as opposed to the *progressive *greedy approach that we introduce in this paper. A modification of this 'direct greedy' approach was also used in our reimplementation DIALIGN-T. Here, we considered not only the weight scores of individual fragments (or their *overlap *weights [1]) but also took into account the overall degree of similarity between the two sequences involved in the fragment. The rationale behind this approach is that a fragment from a sequence pair with high overall similarity is less likely to be a random artefact than a fragment from an otherwise non-related sequence pair, see [16] for details.

### 2.1 Combining segment-based greedy and progressive alignment

To overcome the difficulties of a 'direct' greedy algorithm for multiple alignment, we combined greedy features with a 'progressive' alignment approach [17-20]. Roughly outlined, the new method we developed first computes a *guide tree *for the set of input sequences based on their pairwise similarity scores. The sequences are then aligned in the order defined by the guide tree. We divide the set of fragments contained in the respective optimal pairwise alignments into two subsets *F*_0 _and *F*_1 _where *F*_0 _consists of all fragments with weight scores *below *the average fragment score in all pairwise alignments, and *F*_1 _consists of the fragments with a weight above or equal to the average weight. In a first step, the set *F*_1 _is used to calculate an initial multiple alignment *A*_1 _in a 'progressive' manner. The low-scoring fragments from set *F*_0 _are added later to *A*_1 _in a 'direct' greedy way, provided they are consistent with *A*_1_. In addition, we construct an alternative multiple alignment *A*_0 _using the 'direct' greedy approach implemented in previous versions of DIALIGN and DIALIGN-T, respectively. The program finally returns either *A*_0 _or *A*_1_, depending on which one of these two alignments has the highest score.

To construct a guide tree for the progressive alignment algorithm, we use straight-forward hierarchical clustering. Here, we use a weighted combination of complete-linkage and average-linkage clustering based on pairwise similarity values *R*(*p*, *q*) for pairs of cluster (*C*_*p*_, *C*_*q*_). Initially, each cluster *C*_*i *_consists of one sequence *s*_*i *_only. The similarity *R*(*i*, *j*) between clusters *C*_*i *_and *C*_*j *_(or leaves *i *and *j *in our tree) is defined to be the score of the optimal pairwise alignment of *s*_*i *_and *s*_*j *_according to our objective function, i.e. the sum of the weights of the fragments in an optimal chain of fragments for these two sequences. In every step, we merge the two sequence clusters *C*_*i *_and *C*_*j *_with the maximum similarity value *R*(*i*, *j*) into a new cluster. Whenever a new cluster *C*_*p *_is created by merging clusters *q *and *r *(or a node *p *in the tree is created with children *q *and *r*), we define the similarity between *p *and all other remaining clusters *m *to be

$$R(m,p):=0.1\cdot \frac{1}{2}(R(m,p)+R(m,q))+0.9\cdot \max(R(m,p),R(m,q))$$

The choice of this function has been inspired by MAFFT [21,22]; it also worked very well in our situation on globally and locally related sequences after experiments on BALIBASE 3, BRAliBase II, IRMBASE 2 and DIRMBASE 1.

### 2.2 Merging two sub-alignments

The final multiple alignment of our input sequence set *S *is constructed bottom-up along the guide tree. Thus, the crucial step is to combine two sub-alignments represented by nodes *q *and *r *in our tree whenever a new node *p *is created. In the traditional 'progressive' alignment approach, this is done by calculating a pairwise alignment of *profiles*, but this procedure cannot be directly adapted to our segment-based approach. Let *A*_*q *_and *A*_*r *_be the existing subalignments of the sequences in clusters *C*_*q *_and *C*_*r*_, respectively, at the time where these clusters are merged to a new cluster *C*_*p*_. Let *F*_*q, r *_be the set of all fragments *f *∈ *F *connecting one sequence from cluster *C*_*q *_with another sequence from cluster *C*_*r*_. Now, our main goal is to find a subset *F*_*p*_⊂ *F*_*q*,*r *_with maximum total weight score that is consistent with the existing alignments *A*_*q *_and *A*_*r*_. In other words, we are looking for a subset *F*_*p*_⊂ *F*_*q, r *_with maximum total weight such that

*A*_*p *_= *A*_*q*_∪ *A*_*r*_∪ *F*_*p*_

describes a valid multiple sequence alignment of the sequence set represented by node *p*.

It is easy to see that at this time, before clusters *A*_*q *_and *A*_*r *_are merged, *every single *fragment *f *∈ *F*_*q*,*r *_is consistent with the existing (partial) alignments *A*_*q *_and *A*_*r *_and therefore consistent with the set of all fragments accepted so far. Only *groups *of at least two fragments from *F*_*q*,*r *_can lead to inconsistencies with the previously accepted fragments. Thus, there are different subtypes of consistency conflicts in *F*_*q*,*r *_that may arise when *A*_*q *_and *A*_*r *_are fixed. There are pairs, triples or, in general, *l*-tuples of fragments of *F*_*q*,*r *_that give rise to a conflict in the sense that the conflict can be resolved by removing exactly one fragment of such a conflicting *l*-tuple. Statistically, pairs of conflicting fragments are the most frequent type of conflict, so we will take care of them more intelligently rather than using only a greedy method. Since in our approach, the length of fragments is limited, we can easily determine in constant time for any pair of fragments (*f*_1_, *f*_2_) if the set

*A*_*q*_∪ *A*_*r*_∪ {*f*_1_, *f*_2_}

is consistent, i.e. if it forms a valid alignment, or if there is a pairwise conflict between *f*_1 _and *f*_2_. Here, the data structures described in [23] are used. With unbounded fragment length, the consistency check for the new fragments (*f*_1_, *f*_2_) would take *O*(|*f*_1_| × |*f*_2_|) time where |*f*| is the length of a fragment *f *.

This gives rise to a conflict graph *G*_*q*,*r *_that has a weighted node *n*_*f *_for every fragment *f *∈ *F*_*q*,*r*_. The weight *w*(*n*_*f*_) of node *n*_*f *_is defined to be the weight score *w*(*f*) of *f*, and for any two fragments *f*_1_, *f*_2 _there exists an edge connecting $n_{f_{1}}$ and $n_{f_{2}}$ iff there is a *pairwise conflict *between *f*_1 _and *f*_2_, i.e. if the set *A*_*q*_∪ *A*_*r*_∪ {*f*_1_, *f*_2_} is *inconsistent*. We are now interested in finding a good subset of *F*_*q*,*r *_that does not contain any pairwise conflicts in the above sense. The optimum solution would be obtained by removing a minimal weighted vertex cover from *G*_*q*,*r*_. Since the weighted vertex cover problem is NP-complete we apply the 2-approximation given by Clarkson [24]. This algorithm roughly works as follows: in order to obtain the vertex cover *C*, the algorithm iteratively adds the node *v *with the maximum value

$$\frac{degree(v)}{w(v)}$$

to *C*. For any edge (*v*, *u*) that connects a node *u *with *v *the weight *w*(*u*) is updated to

$$w(u):=w(u)-\frac{degree(v)}{w(v)}$$

and the edge (*u*, *v*) is deleted. This iteration is followed as long as there are edges left.

Note that it is *not *sufficient to remove the vertex cover *C *from *F*_*q*,*r *_to obtain a valid alignment since in the construction of *C*, only inconsistent pairs of fragments were considered. We therefore first remove *C *from *F*_*q*,*r *_and we subsequently remove further inconsistent fragments from *F*_*q*,*r *_using our 'direct' greedy alignment as described in [16]. A consequence of this further reduction of the set *F*_*q*,*r *_is that fragments that were previously removed because of pairwise inconsistencies, may became consistent again. A node $n_{f_{1}}$ may have been included into the set *C *and therefore removed from the alignment as the corresponding fragment *f*_1 _is part of an inconsistent fragment pair (*f*_1_, *f*_2_). However, after subtracting the set *C *from *F*_*q, r*_, the algorithm may detect that fragment *f*_2 _is part of a larger inconsistent group, and *f*_2 _is removed as well. In this case, it may be possible to include *f*_1 _again into the alignment. Therefore, our algorithm reconsiders in a final step the set *C *to see if some of the previously excluded fragments can now be reincluded into the alignment. This is again done using our 'direct' greedy method.

### 2.3 The overall algorithm

In the previous section, we discussed all ingredients that are necessary to give a high-level description of our algorithm to compute a multiple sequence alignment. For clarity, we omit algorithmical details and data structures such as the *consistency frontiers *or *consistency boundaries*, respectively, that are used to check for consistency as these features have been described elsewhere [23]. We use a subroutine *PAIRWISE_ALIGNMENT *(*s*_*i*_, *s*_*j*_, *A*) that takes two sequences *s*_*i *_and *s*_*j *_and (optionally) an existing consistent set of fragments *A *as input and calculates an optimal alignment of *s*_*i *_and *s*_*j *_under the side constraint that this alignment is consistent with *A *and that only those positions in the sequences are aligned that are not yet aligned by a fragment from *A*. Note that in DIALIGN, an alignment is defined as an equivalence relation on the set of all sequence positions, so a consistent set of fragments corresponds to an alignment. Therefore, we do not formally distinguish between alignments and sets of fragments.

**Figure 1:**
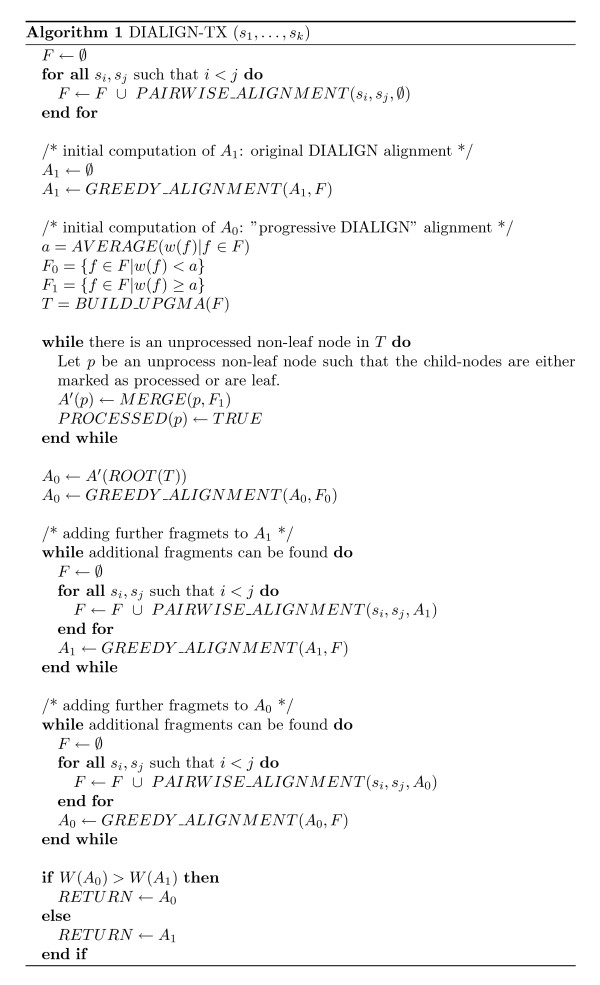
High-level description of our algorithm to calculate a multiple alignment of a set of input sequences *s*_1_, . . ., *s*_*k*_. The algorithm calculates a first alignment *A*_0 _using our novel *progressive *approach and a second alignment *A*_1 _with the greedy method previously used in DIALIGN. Finally, the alignment with the higher numerical score is returned. For the progressive method, *fragments*, i.e. local gap-free pairwise alignments from the respective optimal pairwise alignments are considered. Fragments with a weight score above the average fragment score are processed first following a *guide tree *as described in the main text. Lower-scoring fragments are added later, provided they are consistent with the previously included high-scoring fragments. Note that the output of the sub-routine *PAIRWISE_ALIGNMENT *is a chain of fragments. This is equivalent to a pairwise alignment in the sense of DIALIGN.

Next, a subroutine *GREEDY_ALIGNMENT *(*A*, *F'*) takes an alignment *A *and a set of fragments *F' *as arguments and returns a new alignment *A*' ⊃ *A *by adding fragments from the set *F' *in a 'directly' greedy fashion. For details on these subroutines see also [16]. Furthermore we use a subroutine *BUILD_UPGMA *(*F'*) that takes a set *F' *of fragments as arguments and returns a tree and a subroutine *MERGE*(*p*, *F'*) that takes the parent node *p *and the set of fragments *F' *as argument and returns an alignment of the set of sequences represented by node *p*. Those two subroutines are described in the previous two subsections. A pseudo-code description of the complete algorithm for multiple alignment is given in Figure 1. As in the original version of DIALIGN [1], the process of pairwise alignment and consistency filtering is carried out iteratively. Once a valid alignment *A *has been constructed by removing inconsistent fragments from the set *F' *of the fragments that are part of the respective optimal pairwise alignments, this procedure is repeated until no new fragments can be found. In the second and subsequent iteration steps, only those parts of the sequences are considered that are not yet aligned and optimal pairwise alignments are calculated under the consistency constraints imposed by the existing alignment *A*.

## 3 Further program features

Beside the above described improvements of the optimization algorithm, we incorporated new features into DIALIGN-TX that were already part of the original implementation of DIALIGN. DIALIGN-TX now supports *anchor points *the same way DIALIGN 2.2 does [5,15]. Anchor points can be used for various purposes, e.g. to speed up alignment of large genomic sequences [25,6], or to incorporate information about locally conserved motifs. This can been done, for example, using the *N-local-decoding *approach [26,27] or other methods for motif finding.

DIALIGN-TX also now comes with an option to specify a threshold parameter *T *in order to exclude low-scoring fragments from the alignment. Following an approach proposed in [28], the alignment procedure can be iterated, starting with a high value of *T *and with lower values in subsequent iteration steps. By default, in the first iteration step of our algorithm, we use a value of *T *= -log_2_(0.5) for the pairwise alignment phase, while in all subsequent iteration steps, a value of *T *= 0 is usedd. With a user-specified threshold of *T *= 2 for the first iteration step, the threshold value remains -*log*_2 _(0.5) in all subsequent steps, and with a chosen threshold value of *T *= 1, the value for the subsequent iteration steps is set to -*log*_2 _(0.75).

An optimal pairwise alignment in the sense of our fragment-based approach is a chain of fragments with maximum total weight score. Calculating such an optimal alignment takes *O*(*l*^3^) time if *l *is the (maximum) length of the two sequences since all possible fragments are to be considered. If the length of fragments is bounded by a constant *L*, the complexity is reduced to *O*(*l*^2 ^× *L*). In practice, however, it is not meaningful to consider all possible fragments. Our algorithm processes fragments starting at a pair of positions *i *and *j*, respectively, with increasing fragment length. To reduce the number of fragments considered, our algorithm stops processing longer fragments starting at *i *and *j *if the previously visited short fragments starting at the same positions have low scores. More precisely, we consider the average substitution score of aligned amino acids or the average number of matches for DNA or RNA alignment, respectively, to decide if further fragments starting at *i *and *j *are considered.

To reduce the run time for pairwise alignments, we implemented an option called *fast mode*. This option uses a lower threshold value for the average subsitution scores or number of matches. By default, during the pairwise alignment phase, fragments under consideration are extended until their average substitution score is at least 4 for amino acids (note that our BLOSUM62 matrix has 0 for the lowest score possible) and 0.25 for nucleotides, respectively. With the *fast mode *option, this threshold is increased by 0.25 which has the effect that the extension of fragments during the pairwise alignment phase is interrupted far more often than by default. This option, however, reduces the sensitivity of the program. We observed speed-ups up to factor 10 on various benchmark data when using this option while the alignment quality was still reasonably high, in the sense that the average sum-of-pair score and average column score on our benchmarks deteroriated around 5% – 10% only. We recommend to use this option for large input data containing sequences that are not too distantly related. Hence, this option is not advisable for strictly locally related sequences where we observed a reduction of the alignment quality almost down to a score of zero. However in the latter case this option is not necessary since the original similarity score thresholds of 4 and 0.25, respectively, are effective enough to prevent DIALIGN-TX of unnecessarily looking at too many spurious fragments.

## 4 Benchmark results

In order to evaluate the improvements of the new heuristics we had several benchmarks on various reference sets and compared DIALIGN-TX with its predecessor DIALIGN-T 0.2.2 [16], DIALIGN 2.2 [29], CLUSTAL W2 [30], MUSCLE 3.7 [31], T-COFFEE 5.56 [32] POA V2 [33,34], PROBCONS 1.12 [35] & PROBCONSRNA 1.10, MAFFT 6.240 L-INSi and E-INSi [21,22]. We performed benchmarks for DNA as well as for protein alignment. As globally related benchmark sets we used BRAliBase II [36,37] for RNA and BALIBASE 3 [38] for protein sequences.

The benchmarks on locally related sequence sets were run on IRMBASE 2 for proteins and DIRMBASE 1 for DNA sequences, which have been constructed in a very similar way as IRMBASE 1 [16] by implanting highly conserved motifs generated by ROSE [39] in long random sequences. IRMBASE 2 and DIRM-BASE 1 both consist of four reference sets ref1, ref2, ref3 and ref4 with one, two, three and four (respectively) randomly implanted ROSE motives. The major difference compared to the old IRMBASE 1 lies in the fact that in 1/*s *cases the occurrence of a motive in a sequence has been omitted randomly, whereby *s *is the number of sequences in the sequence family. The results on IRMBASE 2 and DIRMBASE 1 now tell us how the alignment programs perform in cases when it is unknown if every motive occurs in every sequence thus providing a more realistic basis for assessing the alignment quality on locally related sequences compared to the situation in the old IRMBASE 1 where every motive *always *occurred in *every *sequence.

Each reference set in IRMBASE 2 and DIRMBASE 1 consists of 48 sequence families, 24 of which contain ROSE motifs of length 30 while the remaining families contain motifs of length 60. 16 sequence families in each of the reference sets consist of 4 sequences each, another 16 families consist of 8 sequences while the remaining 16 families consist of 16 sequences. In ref1, random sequences of length 400 are added to the conserved ROSE motif while for ref2 and ref3, random seqences of length 500 are added. In ref4 random sequences of length 600 are added.

For both BAliBASE and IRMBASE, we used two different criteria to evaluate multi-alignment software tools. We used the *sum-of-pair score *where the percentage of correctly aligned *pairs *of residues is taken as a quality measure for alignments. In addition, we used the *column score *where the percentage of correct *columns *in an alignment is the criterion for alignment quality. Both scoring schemes were restricted to *core blocks *within the reference sequences where the 'true' alignment is known. For IRMBASE 2 and DIRMBASE 1, the core blocks are defined as the conserved ROSE motifs. To compare the output of different programs to the respective benchmark alignments, we used C. Notredame's program aln_compare [32].

### 4.1 Results on locally related sequence families

The quality results of our benchmarks of DIALIGN-TX and various alignment programs on the local aligment databases can be found in Tables 1 and 2 for the local protein database IRMBASE 2 and in Tables 3 and 4 for the local DNA database DIRMBASE 1. The average CPU times of the tested methods are listed in Table 5. When looking at the results DIALIGN-TX clearly outperforms all other methods on sum-of-pairs score (SPS) and column score (CS) with the only exception that MAFFT E-INSi outperforms DIALIGN-TX on the SPS on IRMBASE 2 whilst in turn DIALIGN-TX is around 3.5 times faster and significantly outperforms MAFFT-EINSi on the CS. The superiority of DIALIGN-TX compared to DIALIGN-T 0.2.2 is not statistically significant on IRMBASE 2, however it is on DIRMBASE 1 which is due to a very low sensitivity threshold parameter for the DNA case set by default in DIALIGN-T 0.2.2 that allowed fragments solely comprised of matches. In *all *other comparisons DIALIGN-TX is significantly superior to the other programs with respect to the Wilcoxon Matched Pairs Signed Rank Test [40]. DIALIGN 2.2, DIALIGN-T 0.2.2 (only for protein), MAFFT L-INSi and MAFFT E-INSi were the only other methods that produced reasonable results.

**Table 1:** Sum-of-pairs scores of various alignment programs on the benchmark database IRMBASE 2

Method (Protein)	REF1	REF2	REF3	REF4	Total
DIALIGN-TX	89.42	**94.90**	93.75	93.64	92.93
DIALIGN-T 0.2.2	89.67^0^	94.19^0^	**93.93**^0^	93.12^0^	92.73^0^
DIALIGN 2.2	90.43^0^	93.40^-^	91.78^--^	92.98^-^	92.15^--^
CLUSTAL W2	07.13^--^	10.63^--^	19.87^--^	26.17^--^	15.95^--^
T-COFFEE 5.56	72.67^--^	77.80^--^	83.03^--^	83.48^-^	79.24^--^
POA V2	87.56^-^	49.57^--^	41.90^--^	37.56^--^	54.15^--^
MAFFT 6.240 L-INSi	82.78^0^	84.29^-^	84.15^--^	82.42^--^	84.41^--^
MAFFT 6.240 E-INSi	**90.53**^0^	94.37^0^	93.11^0^	**94.79**^+^	**93.20**^+^
MUSCLE 3.7	32.67^--^	34.82^--^	54.19^--^	57.84^--^	44.88^--^
PROBCONS 1.12	78.78^--^	86.82^--^	87.29^-^	87.69^--^	85.15^--^

Average sum-of-pair scores (SPS) of the benchmarked programs on the core blocks (given by the implanted conserved motifs) of IRMBASE 2. *Minus *symbols denote statistically significant *inferiority *of the respective method compared with DIALIGN-TX, while *plus *symbols denote statistically significant superiority of the method. ^0 ^denotes non-significant superiority or inferiority of DIALIGN-TX, respectively. Single plus or minus symbols denote significance according to the Wilcoxon Matched Pairs Signed Rank Test with *p *≤ 0.05 and double symbols denote significance with *p *≤ 0.001, respectively.

**Table 2:** Column scores of different programs on IRMBASE 2

Method (Protein)	REF1	REF2	REF3	REF4	Total
DIALIGN-TX	64.17	**77.36**	70.30	**72.23**	**71.02**
DIALIGN-T 0.2.2	67.04^0^	75.81^0^	70.40^0^	**70.44**^0^	70.93^0^
DIALIGN 2.2	**68.52**^0^	73.32^-^	65.34^-^	69.50^-^	69.17^--^
CLUSTAL W2	00.00^--^	00.00^--^	00.11^--^	02.86^--^	00.74^--^
T-COFFEE 5.56	34.84^--^	40.87^--^	43.62^--^	49.56^--^	42.22^--^
POA V2	50.99^-^	16.95^--^	11.79^--^	10.18^--^	22.47^--^
MAFFT 6.240 L-INSi	37.81^--^	39.54^--^	32.79^--^	38.75^--^	32.22^--^
MAFFT 6.240 E-INSi	45.70^-^	52.37^--^	43.11^--^	54.82^--^	49.00^--^
MUSCLE 3.7	04.65^--^	06.87^--^	14.80^--^	19.65^--^	11.49^--^
PROBCONS 1.12	36.77^--^	43.47^--^	41.89^--^	43.56^--^	41.42^--^

Average column scores (CS) of the benchmarked programs on the core blocks of IRM-BASE 2. The symbols are analogous to Table 1.

**Table 3:** Sum-of-pairs scores on DIRMBASE 1

Method (DNA)	REF1	REF2	REF3	REF4	Total
DIALIGN-TX	**94.38**	**92.85**	**95.44**	**95.70**	**94.59**
DIALIGN-T 0.2.2	64.00^--^	61.22^--^	64.96^--^	65.24^--^	63.85^--^
DIALIGN 2.2	92.61^-^	91.10^-^	94.62-	94.13^-^	93.12^--^
CLUSTAL W2	06.79^--^	08.27^--^	18.51^--^	29.09^--^	15.66^--^
T-COFFEE 5.56	14.71^--^	18.88^--^	32.08^--^	43.39^--^	27.62^--^
POA V2	32.03^--^	27.40^--^	28.78^--^	32.18^--^	30.10^--^
MAFFT 6.240 L-INSi	52.40^--^	48.81^--^	49.77^--^	57.47^--^	52.36^--^
MAFFT 6.240 E-INSi	92.42^0^	84.15^--^	87.91^-^	89.36^-^	88.46^--^
MUSCLE 3.7	48.17^--^	54.40^--^	56.57^--^	60.24^--^	56.84^--^
PROBCONSRNA 1.10	13.00^--^	12.94^--^	20.28^--^	32.56^--^	19.69^--^

Average sum-of-pair scores (SPS) of the benchmarked programs on the core blocks of DIRMBASE 1. The symbols are analogous to Table 1.

**Table 4:** Column scores on DIRMBASE 1.

Method (DNA)	REF1	REF2	REF3	REF4	Total
DIALIGN-TX	**74.39**	**69.03**	**71.57**	**75.11**	**72.52**
DIALIGN-T 0.2.2	29.60^--^	28.63^--^	35.51^--^	35.85^--^	32.40^--^
DIALIGN 2.2	69.95^0^	68.19^0^	71.25^0^	72.48^0^	70.47^-^
CLUSTAL W2	00.00^--^	00.00^--^	02.19^--^	04.99^--^	01.80^--^
T-COFFEE 5.56	00.00^--^	00.18^--^	04.01^--^	08.44^--^	03.16^--^
POA V2	05.63^--^	07.32^--^	04.12^--^	06.81^--^	05.97^--^
MAFFT 6.240 L-INSi	21.45^--^	11.93^--^	16.02^--^	22.30^--^	17.93^--^
MAFFT 6.240 E-INSi	40.28^--^	41.99^--^	45.77^--^	51.01^--^	44.76^--^
MUSCLE 3.7	14.18^--^	16.18^--^	19.62^--^	30.43^--^	20.10^--^
PROBCONSRNA 1.10	00.73^--^	00.05^--^	01.34^--^	04.31^--^	01.61^--^

Average column scores (CS) of the benchmarked programs on the core blocks of DIRM-BASE 1. The symbols are analogous to Table 1.

**Table 5:** Program run time on IRMBASE 2 and DIRMBASE 1

Method	Average runtime on IRMBASE 2	Average runtime on DIRMBASE 1
DIALIGN-TX 1.0	4.47	9.84
DIALIGN-T 0.2.2	2.73	2.31
DIALIGN 2.2	4.98	4.82
CLUSTAL W2	1.86	1.36
T-COFFEE 5.56	26.41	365.88
POA V2	1.81	1.20
MAFFT 6.240 L-INSi	8.47	5.33
MAFFT 6.240 E-INSi	15.35	8.39
MUSCLE 3.7	6.34	4.87
PROBCONS(RNA) 1.12(1.10)	28.27	18.54

Average running time (in seconds) per multiple alignment for sequence families on IRMBASE 2 and DIRMBASE 1. Program runs were performed on a Linux workstation with an 3.2 GHz Pentium 4 processor and 2 GB RAM.

On IRMBASE 2 our new program DIALIGN-TX is around 1.64 times slower compared to DIALIGN-T however it is still faster than DIALIGN 2.2, on DIRM-BASE 1 we observed that DIALIGN-TX is 4.26 times slower than DIALIGN-T (which is due to the reduced sensitivity in DIALIGN-T 0.2.2) and we also see that DIALIGN-TX is around 2.04 slower than DIALIGN 2.2. Although IRMBASE 2 and DIRMBASE 1 are constructed in a similar way we see that T-COFFEE and PROBCONS behave quite well on the protein alignments whereas the perform very poorly in the DNA case while the other methods ranked mostly equal in the protein and DNA case. Overall, we conclude from our benchmarks that DIALIGN-TX is the dominant program on locally related sequence protein and DNA families that consist of closely related motives embedded in long unalignable sequences.

### 4.2 Results on globally related sequence families

The results of our benchmark on the global alignment databases are listed in the Tables 6, 7 for BALIBASE 3 and in Tables 8, 9 for core blocks of BRAliBase II. The average CPU times of all methods can be found in Table 10. According to the Wilcoxon Matched Pairs Signed Rank Test DIALIGN-TX outperforms DIALIGN-T 0.2.2, DIALIGN 2.2, POA and CLUSTAL W2 on BALIBASE3 whereby DIALIGN-TX is the only method following the DIALIGN approach that significantly outperforms CLUSTAL W2. Since the methods T-COFFEE, PROBCONS, MAFFT and MUSCLE are focused on global alignments, they significantly outperform DIALIGN-TX on BALIBASE 3. Overall PROBCONS, MAFFT L-INSi and E-INSi are the superior methods on BALIBASE 3. On BALIBASE 3 the new DIALIGN-TX program is around 1.22 times slower than the previous version of DIALIGN-T and around 1.36 times faster than DIALIGN 2.2.

**Table 6:** Sum-of-pairs scores on BALIBASE 3

Method (Protein)	RV11	RV12	RV20	RV30	RV40	RV50	Total
DIALIGN-TX	51.52	89.18	87.87	76.18	83.65	82.28	78.83
DIALIGN-T 0.2.2	49.30^-^	88.76^0^	86.29^0^	74.66^0^	81.95^-^	80.14^-^	77.31^--^
DIALIGN 2.2	50.73^0^	86.66^-^	86.91^0^	74.05^0^	83.31^0^	80.69^0^	77.52^--^
CLUSTAL W2	50.06^0^	86.43^0^	85.16^0^	72.50^-^	78.93^0^	74.24^-^	75.36^--^
T-COFFEE 5.56	58.22^++^	92.27^++^	90.92^++^	79.09^+^	86.03^+^	86.09^+^	82.41^++^
POA V2	37.96^--^	83.19^--^	85.28^-^	71.93^-^	78.22^--^	71.49^--^	72.17^--^
MAFFT 6.240 L-INSi	**67.11**^++^	93.63^++^	**92.67**^++^	85.55^++^	**91.97**^++^	**90.00**^++^	**87.07**^++^
MAFFT 6.240 E-INSi	66.00^++^	93.61^++^	92.64^++^	**86.12**^++^	91.46^++^	89.91^++^	86.83^++^
MUSCLE 3.7	57.90^+^	91.67^++^	89.17^+^	80.60^+^	87.26^+^	83.39^0^	82.19^++^
PROBCONS 1.12	66.99^++^	**94.12**^++^	91.68^++^	84.61^++^	90.24^++^	89.28^++^	86.40^++^

Average sum-of-pair scores (SPS) of the benchmarked programs on the core blocks of BALIBASE 3. The symbols are analogous to Table 1.

**Table 7:** Column scores on BALIBASE 3

Method (Protein)	RV11	RV12	RV20	RV30	RV40	RV50	Total
DIALIGN-TX 1.0	26.53	75.23	30.49	38.53	44.82	46.56	44.34
DIALIGN-T 0.2.2	25.32^0^	72.55^0^	29.20^0^	34.90^-^	45.23^0^	44.25^0^	42.76^-^
DIALIGN 2.2	26.50^0^	69.55^-^	29.22^0^	31.23^-^	44.12^0^	42.50^-^	41.49^--^
CLUSTAL W2	22.74^0^	71.59^0^	21.98^0^	27.23^-^	39.55^0^	30.75^-^	37.35^--^
T-COFFEE 5.56	31.34^0^	81.18^++^	37.81^+^	36.57^0^	48.20^0^	50.63^0^	48.54^++^
POA V2	15.26^--^	63.84^--^	23.34^-^	28.23^-^	33.67^--^	27.00^--^	33.37^--^
MAFFT 6.240 L-INSi	**44.61**^++^	83.75^++^	**45.27**^++^	56.93^++^	**59.69**^++^	56.19^+^	**58.57**^++^
MAFFT 6.240 E-INSi	43.71^++^	83.43^++^	44.63^++^	**58.80**^++^	58.33^++^	**58.94**^++^	58.37^++^
MUSCLE 3.7	33.03^+^	80.46^++^	35.22^0^	38.77^0^	45.96^0^	44.94^0^	47.58^++^
PROBCONS 1.12	41.68^++^	**85.52**^++^	40.49^++^	54.37^++^	52.90^++^	56.50^++^	55.66^++^

Average column scores (CS) of the benchmarked programs on the core blocks of BAL-IBASE 3. The symbols are analogous to Table 1.

**Table 8:** Sum-of-pairs scores on BRAliBase II

Method (DNA)	G2In	rRNA	SRP	tRNA	U5	Total
DIALIGN-TX 1.0	72.08	91.69	82.92	78.53	77.80	80.42
DIALIGN-T 0.2.2	54.68^--^	69.13^--^	60.81^--^	64.44^--^	67.87^--^	63.53^--^
DIALIGN 2.2	71.72^0^	89.89^--^	81.47^--^	78.57^0^	76.16^--^	79.37^--^
CLUSTAL W2	72.68^0^	93.25^+^	87.40^++^	86.96^++^	79.56^+^	83.80^++^
T-COFFEE 5.56	73.79^0^	90.94^+^	83.90^0^	81.65^0^	79.13^+^	81.73^+^
POA V2	67.22^--^	88.92^--^	85.47^++^	76.91^-^	77.28^0^	79.02^--^
MAFFT 6.240 L-INSi	78.93^++^	93.85^+^	87.46^++^	91.79^++^	82.80^++^	86.84^++^
MAFFT 6.240 E-INSi	77.39^++^	93.80^+^	87.24^++^	90.60^++^	80.46^++^	85.71^++^
MUSCLE 3.7	76.42^++^	94.04^+^	87.06^++^	87.27^++^	79.71^+^	84.69^++^
PROBCONSRNA 1.10	**80.08**^++^	**94.48**^++^	**88.07**^++^	**92.58**^++^	**84.76**^++^	**87.90**^++^

Average sum-of-pair scores (SPS) of the benchmarked programs on BRAliBase II. The The symbols are analogous to Table 1.

**Table 9:** Column scores on BRAliBase II

Method (DNA)	G2In	rRNA	SRP	tRNA	U5	Total
DIALIGN-TX 1.0	60.85	84.33	70.95	68.05	62.71	69.03
DIALIGN-T 0.2.2	36.51^--^	50.00^--^	42.34^--^	52.01^--^	50.34^--^	46.43^--^
DIALIGN 2.2	60.90^0^	81.08^--^	68.53^--^	67.59^0^	60.11^-^	67.29^--^
CLUSTAL W2	61.24^0^	86.72^0^	76.61^++^	76.20^++^	65.11^+^	72.85^++^
T-COFFEE 5.56	60.24^0^	82.56^-^	71.63^0^	69.23^0^	62.93^0^	69.01^0^
POA V2	55.21^--^	80.38^--^	73.77^++^	66.03^0^	61.63^0^	67.12^--^
MAFFT 6.240 L-INSi	65.23^+^	87.49^+^	76.75^++^	84.59^++^	68.46^++^	76.25^++^
MAFFT 6.240 E-INSi	63.84^+^	87.34^+^	76.59^++^	83.29^++^	65.71^++^	75.04^++^
MUSCLE 3.7	63.20^0^	87.97^+^	76.57^++^	78.01^++^	64.34^+^	73.64^++^
PROBCONSRNA 1.10	**68.70**^++^	**88.60**^++^	**77.55**^++^	**85.46**^++^	**71.73**^++^	**78.19**^++^

Average column scores (CS) of the benchmarked programs on BRAliBase II. The symbols are analogous to Table 1.

**Table 10:** Run time on BALIBASE 3 and BRAliBase II

Method	Average runtime on BALIBASE 3	Average runtime on BRAliBase II
DIALIGN-TX 1.0	33.37	0.15
DIALIGN-T 0.2.2	27.79	0.08
DIALIGN 2.2	45.41	0.09
CLUSTAL W2	8.72	0.07
T-COFFEE 5.56	315.78	1.95
POA V2	8.07	0.04
MAFFT 6.240 L-INSi	19.51	0.26
MAFFT 6.240 E-INSi	28.26	0.27
MUSCLE 3.7	10.49	0.05
PROBCONS(RNA) 1.12(1.10)	168.65	0.24

Average running time (in seconds) per multiple alignment for sequence families on BALIBASE 3 and BRAliBase II. Program runs were performed on a Linux workstation with an 3.2 GHz Pentium 4 processor and 2 GB RAM.

We have a slightly different picture in the RNA case we examined using BRAliBase II benchmark database that has an even stronger global character and is the only benchmark database that we used that does not come with core blocks. DIALIGN-TX significantly outperforms POA and all other versions of DIALIGN approach whereas it is still inferior to the global methods CLUSTAL W2, MAFFT, MUSCLE and PROBCONSRNA. The difference between T-COFFEE and DIALIGN-TX on BRAliBase II is quite small, i.e. T-COFFEE outperforms DIALIGN-TX only on the SPS whereas there is no significant different on the CS. Since MAFFT and PROBCONSRNA have been trained on BRAliBase II the dominance of those methods (especially PROBCONSRNA) is not very surprising. Regarding CPU time DIALIGN-TX is approximately 1.7 times slower than DIALIGN-T 0.2.2 and DIALIGN 2.2 on BRAliBase II.

## 5 Conclusion

In this paper, we introduced a new optimization algorithm for the segment-based multiple-alignment problem. Since the first release of the program DIALIGN in 1996, a 'direct' greedy approach has been used where local pairwise alignments (fragments) are checked for consistency one-by-one to see if they can be included into a valid multiple alignment. In this approach, the order in which fragments are checked for consistency is basically determined by their individual *weight scores*. Some modifications have been introduced, such as *overlap weights *[1] and a more context-sensitive approach that takes into account the overal significance of the pairwise alignment to which a fragment belongs [16]. Nevertheless, a 'direct' greedy approach is always sensitive to spurious pairwise random similarities and may lead to alignments with scores far below the possible optimal score (e.g. [13,5], Pöhler and Morgenstern, unpublished data).

The optimization method that we introduced herein is inspired by the so-called *progressive *approach to multiple alignment introduced in the 1980s for the classical multiple-alignment problem [17]. We adapted this alignment strategy to our segment-based approach using an existing graph-theoretical optimization algorithm and combined it with our previous 'direct' greedy approach. As a result, we obtain a new version of our program that achieves significantly better results than the previous versions of the program, DIALIGN 2 and DIALIGN-T.

To test our method, we used standard benchmark databases for multiple alignment of protein and nucleic-acid sequences. Since these databases are heavily biased towards *global *alignment, we also used a benchmark database with simulated local homologies. The test results on these data confirm some of the known results on the performance of multiple-alignment programs. On the globally related sequence sets from BAliBASE and BRALIBASE, the segment-based approach is outperformed by classical, strictly global alignment methods. However, even on these data, we could achieve a considerable improvement with the new optimization algorithm used in DIALIGN-TX. On the simulated local homologies, our method clearly outperforms other alignment approaches, and again the new algorithm introduced in this paper achieved significantly better results than older versions of DIALIGN. Among the methods for global multiple alignment, the program MAFFT [21,22] performed remarkably well, not only on globally, but also on locally related sequences.

We will conduct further studies to investigate to what extent the optimization algorithms used in the different versions of DIALIGN can be improved and which alternative algorithms can be applied to the optimization problem given by the segment-based alignment approach.

## Program availability

DIALIGN-TX is online available at *Göttingen Bioinformatics Compute Server (**GOBICS**) *at [41] The program source code and our benchmark databases IRMBASE 2 and DIRMBASE 1 are downloadable from the same web site.

## Authors' contributions

ARS conceived the new methods, implemented the program, constructed IRM-BASE 2 and DIRMBASE 1, did the evaluation and wrote parts of the manuscript, MK and BM supervised the work, provided resources and wrote parts of the manuscript. All authors read and approved the final manuscript.
